# Sevoflurane and Hypercapnia Blunt the Physiological Variability of Spontaneous Breathing: A Comparative Interventional Study

**DOI:** 10.3389/fphys.2022.871070

**Published:** 2022-04-11

**Authors:** Adam L. Balogh, Roberta Sudy, Ferenc Petak, Walid Habre, Andre Dos Santos Rocha

**Affiliations:** ^1^ Unit for Anaesthesiological Investigations, Department of Acute Medicine, University of Geneva, Geneva, Switzerland; ^2^ Department of Medical Physics and Informatics, University of Szeged, Szeged, Hungary

**Keywords:** breathing variability, rabbit, wakefulness, sleep, sedation, whole-body plethysmography, tidal volume, inspiratory time

## Abstract

**Background:** Although spontaneous breathing is known to exhibit substantial physiological fluctuation that contributes to alveolar recruitment, changes in the variability of the respiratory pattern following inhalation of carbon dioxide (CO_2_) and volatile anesthetics have not been characterized. Therefore, we aimed at comparing the indices of breathing variability under wakefulness, sleep, hypercapnia and sedative and anesthetic concentrations of sevoflurane.

**Methods:** Spontaneous breathing pattern was recorded on two consecutive days in six rabbits using open whole-body plethysmography under wakefulness and spontaneous sleep and following inhalation of 5% CO_2_, 2% sevoflurane (0.5 MAC) and 4% (1 MAC) sevoflurane. Tidal volume (V_T_), respiratory rate (RR), minute ventilation (MV), inspiratory time (T_I_) and mean inspiratory flow (V_T_/T_I_) were calculated from the pressure fluctuations in the plethysmograph. Means and coefficients of variation were calculated for each measured variable. Autoregressive model fitting was applied to estimate the relative contributions of random, correlated, and oscillatory behavior to the total variance.

**Results:** Physiological sleep decreased MV by lowering RR without affecting V_T_. Hypercapnia increased MV by elevating V_T_. Sedative and anesthetic concentrations of sevoflurane increased V_T_ but decreased MV due to a decrease in RR. Compared to the awake stage, CO_2_ had no effect on V_T_/T_I_ while sevoflurane depressed significantly the mean inspiratory flow. Compared to wakefulness, the variability in V_T_, RR, MV, T_I_ and V_T_/T_I_ were not affected by sleep but were all significantly decreased by CO_2_ and sevoflurane. The variance of T_I_ originating from correlated behavior was significantly decreased by both concentrations of sevoflurane compared to the awake and asleep conditions.

**Conclusions:** The variability of spontaneous breathing during physiological sleep and sevoflurane-induced anesthesia differed fundamentally, with the volatile agent diminishing markedly the fluctuations in respiratory volume, inspiratory airflow and breathing frequency. These findings may suggest the increased risk of lung derecruitment during procedures under sevoflurane in which spontaneous breathing is maintained.

## BackGround

The respiratory system demonstrates appreciable tidal variation in rate and volume, even at rest and in steady-state conditions (Dejours et al., 1966; Frey et al., 1998). Aside from the constant adjustment to internal and external conditions, these fluctuations in the breathing pattern are beneficial for structural and functional reasons, given the nonlinear behavior of the lungs (Suki et al., 1998). Accordingly, variability in tidal volume (V_T_) and respiratory rate (RR) promotes alveolar recruitment and optimizes ventilation-perfusion matching (Ma et al., 2011; Huhle et al., 2016). Thus, the loss or diminishment of breathing variability may lead to atelectasis and increased shunt resulting in deterioration of respiratory mechanics and gas exchange.

Spontaneous breathing is often maintained during procedures performed under sedation in anesthesia and intensive care settings. However, the variability of spontaneous breathing is affected by various sedative drugs (Klimathianaki et al., 2010; Bradley et al., 2013), which may impact gas exchange and post-procedural outcomes. In addition, sedation may lead to hypercapnia, the major contributor of the control of breathing, which by itself influences the variability of spontaneous breathing (Fiamma et al., 2007). In children sevoflurane is commonly used (Brioni et al., 2017) thanks to its tolerable odor and advantageous pharmacokinetic profile to provide sedation or anesthesia for longer diagnostic procedures or certain surgical interventions. Although the effects of different anesthetic agents and hypercapnia on the mean values of respiratory variables measured during spontaneous breathing have been well characterized (Doi and Ikeda, 1987; Goodman et al., 1987), there is still a lack of knowledge on the effects of these interventions on the variability of spontaneous breathing outcomes, in particular under inhalation anesthesia.

Therefore, the aim of this experimental study was to reveal the changes in the variability of spontaneous breathing under hypercapnia and sevoflurane at concentrations used routinely for sedation and anesthesia. We hypothesized that the significant changes in the spontaneous breathing parameters occurring under hypercapnia and sevoflurane will also manifest as altered variability. Furthermore, we aimed at identifying the contribution of the random and correlated components to the total variance of spontaneous breathing indices.

## Methods

### Ethical Approval

Approval was obtained from the Experimental Ethics Committee of the University of Geneva and the Animal Welfare Committee of the Canton of Geneva, Switzerland (No.GE17/151). Procedures were performed in compliance with all current animal protection laws of Switzerland (LPA, RS455) and reported according to the ARRIVE (Animal Research: Reporting of *in vivo* Experiments) guidelines.

### Experimental Animals

Six adult female New Zealand White rabbits (mean weight 2.60 kg, range 2.40–2.85 kg) were purchased from the farm of the University of Geneva (Arare, Geneva, Switzerland). Food and water were provided *ad libitum* before the experiment, and welfare of the animals was ensured according to the current animal protection laws of Switzerland.

### Open Barometric Whole-Body Plethysmography Measurements

Spontaneous breathing was recorded using a custom-made plexiglass chamber as an open barometric whole-body plethysmograph (WBP). The external measures of the chamber were 300 × 300 × 500 mm, with a wall thickness of 10 mm, resulting in an internal volume of 37.63 L. The chamber pressure was continuously measured with a pressure transducer (Honeywell Differential Pressure Sensor model 24PCEFA6D, Charlotte, North Carolina, United States). The WBP chamber was continuously flushed with a fresh supply of gases to prevent carbon dioxide (CO_2_) accumulation. Humidity and temperature were continuously recorded using a hygrometer and temperature probe (Harvard Apparatus Homeothermic system 230 VAC, Les Ulis, France). The movement of the animals was constantly monitored by a camera. All signals were continuously recorded at a sampling rate of 1 kHz (ADInstruments, Powerlab model 8/35 and LabChart 7, Dunedin, New Zealand).

### Data Analysis

The Peak Analysis extension of the LabChart software was used to identify each breath. Segments with pressure artefacts related to animal movement were excluded from the analysis. Thus, the chamber pressure was analyzed during motionless periods for each condition. Respiratory cycles were defined as segments between two consecutive peak values identified as local maxima in chamber pressure greater than 1.5 times the standard deviation of the entire signal. RR was calculated by identifying peak maxima and minima in chamber pressure corresponding to end-inspiratory and end-expiratory states, respectively. Inspiratory time (T_I_) was defined as the time elapsed from end-expiration to the subsequent end-inspiration. Expiratory time (T_E_) was calculated by subtracting T_I_ from the total length of the respiratory cycle. Expiratory V_T_ was obtained by multiplying the amplitude of chamber pressure by the calibration factor (see below). Mean inspiratory flow was calculated as V_T_/T_I_. Minute volume (MV) was obtained as the product of V_T_ and RR.

### Calculation of Respiratory Volumes

To estimate V_T_ using WBP, tracheal airflow and chamber pressure were measured simultaneously in one representative rabbit. Following anesthesia with intramuscular xylazine (3 mg/kg) and ketamine (25 mg/kg) and supplemental analgesia with subcutaneous lidocaine (1 ml, 0.5%), surgical tracheostomy was performed, and a 3.5 mm internal diameter uncuffed tube was placed in the trachea. A pneumotachograph (Hans Rudolph 8410A, Kansas, United States) was connected to the tracheal tube and the spontaneously breathing rabbit was placed inside the WBP chamber. Expiratory V_T_ was calculated by integration of the expiratory tracheal flow. The calibration factor used to estimate the expiratory V_T_, was determined from 20 consecutive breaths by dividing the measured V_T_ of each breath by the amplitude of the change in the corresponding chamber pressure.

### Study Protocol

Animals were allowed to acclimatize to the measurement environment for one week. Data collection of the breathing pattern was then performed on two consecutive days. On each experimental day, the rabbits were placed in the WBP chamber for 1.5 h in the same order. Five periods of 15 min were sequentially recorded without human presence in the measurement room, with lights off and absence of noise stimuli. During the first 30 min, the box was filled with air (21% oxygen) and measurements were made while the animals were awake (stage Awake). Left alone and unstimulated during this period, all animals progressively adopted a motionless, lay down or sphinx-like posture, depression of the ears and ceased nostril movements. According to rabbit behavioural studies (Pivik et al., 1986; Aguilar-Roblero and González-Mariscal, 2020), this stage likely corresponds to physiological sleep (stage Asleep) and was analyzed separately from the Awake stage. The box was then filled with 5% carbon dioxide and 95% air (stage CO_2_) for 15 min. Finally, after the CO_2_ was removed, 2% sevoflurane corresponding to approximately 0.5 minimum alveolar concentration (MAC) (stage Sev 2%) followed by 4% sevoflurane corresponding to approximately 1 MAC (stage Sev 4%) (Scheller et al., 1988) was introduced into the WBP chamber for 15 min each. These concentrations of sevoflurane were chosen as they induce two measurably different depths of anesthesia, sedation, and general anesthesia in rabbits, respectively (Terada et al., 2014). At concentrations significantly below 0.5 MAC, awakening is expected, while concentrations above 1.3 MAC are rarely used in the clinical environment due to hemodynamic side effects and lack of additional anesthetic effect. All gases were delivered at a flow rate of 1.5 L/min into the chamber, and the gas composition was continuously measured by side-stream gas monitoring (Dräger Vamos, Liebefeld, Switzerland). The rabbits were returned to the housing facility after regaining consciousness.

### Coefficients of Variation, Autocorrelation, and Spectral Analyses

Coefficients of variation (CV) of V_T_, RR, T_I_, T_E_, MV and V_T_/T_I_ were calculated for each protocol stage as the ratio of the standard deviation and the mean for each animal.

For the autocorrelation and spectral analyses, series containing equal number of consecutive breaths were selected for each animal for all stages, and V_T_, T_I_, T_E_ and V_T_/T_I_ were calculated for each breath within the sequence. Since outliers can introduce a substantial bias into the correlogram, values were clamped at ± 2 standard deviations from the mean of each series (Pack et al., 1988). The stationarity of the data series was ensured by third degree polynomial detrending (Jubran et al., 1997). Subsequently, autocorrelation coefficients at a lag of one breath were calculated for each series.

To assess the relative contribution of different components, the gross variance of breath-by-breath data series of V_T_, T_I_, T_E_ and V_T_/T_I_ was partitioned into correlated, random and oscillatory behavior by fitting a previously validated autoregressive model to the data series (Modarreszadeh et al., 1990). Briefly, autoregressive models were fitted to the clamped, detrended data series. The models included a random noise component and a first order autoregressive component if the lag of one breath autocorrelation coefficient was non-significant. If the first order autocorrelation coefficient was significant, a second order autoregressive component was included in the model. If any significant peaks (higher than mean + 2 standard deviations, separately for frequencies below 0.2/breath and above 0.2/breath) were identified in the power spectra of V_T_, T_I_, T_E_ and V_T_/T_I_ data series obtained by fast Fourier transform, an oscillatory component was also included in the model as an external regressor. The autocorrelation coefficients at a lag of one breath of each measurement were categorized as positive or negative. Autocorrelations and spectral analyses were performed in the *R* environment, and the *forecast* package (Hyndman and Khandakar, 2008) was used for the autoregressive model fitting.

### Statistical Analysis

All data are presented as mean [95% confidence interval of the mean], unless otherwise stated. No difference was observed in any of the parameters obtained between the two experimental days, and thus pooled data sets were used in the statistical analysis. One-way repeated measures analyses of variance (ANOVA) were performed for continuous variables to compare means at the five protocol stages with a composite of subject and day as the repeating factor. In case of normality, post hoc pairwise comparisons were performed using Student’s t-tests with Holm–Sidak correction to compare means of absolute values of the parameters and their CVs between the protocol stages. Subsequently, chi-square tests followed by corrected pairwise comparisons were used to analyze the differences in the frequencies of the categorized autocorrelation coefficients. Sample size analysis was based on considering a 20% change in the primary outcome variable (CV of V_T_) as clinically significant. This analysis revealed the need for a minimum of six rabbits to detect statistically significant differences using repeated measures ANOVA on the main outcome variable and assuming a standard deviation of 20%, a power of 0.9 and an alpha level of 0.05. *p* values < 0.05 were considered significant. Statistical analyses were performed with SigmaPlot 13.0 (Systat Software, San Jose, CA) and in the *R* statistical environment.

## Results

**FIGURE 1:**
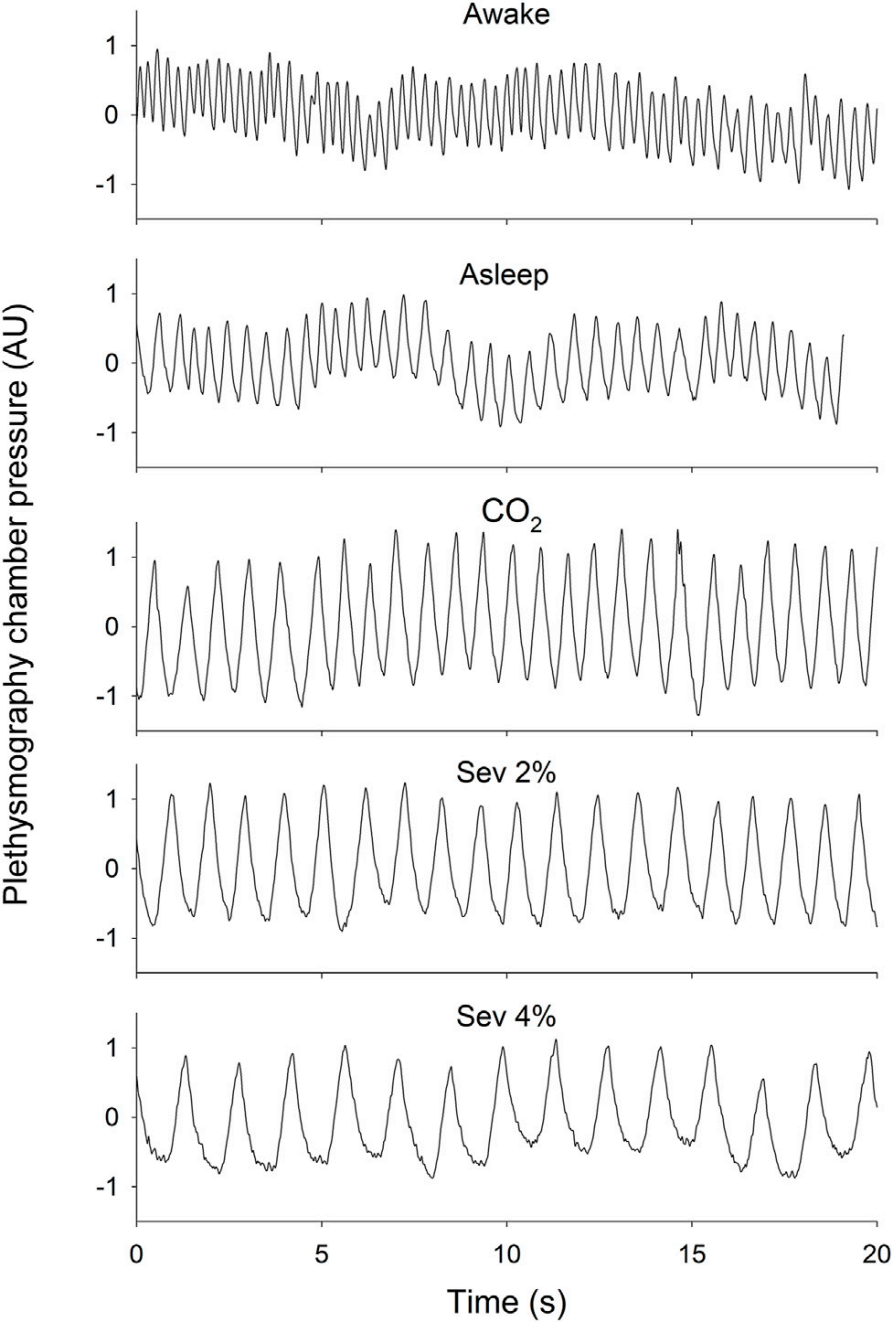
Representative 20-s tracings of plethysmography chamber pressure in wakeful (Awake) and sleeping (Asleep) states, and during the inhalation of 5% CO_2_ (CO_2_), 2% sevoflurane (Sev 2%) or 4% sevoflurane (Sev 4%) with the baseline pressure adjusted to 0. Scaling of the y-axes are identical.

Measurements of chamber pressure did not require correction for changes in chamber temperature [19°C (18.9–19.2°C)] or humidity [33% (32.4–33.7%)], as these were negligible between and within animals during the 1.5-h recordings. Representative tracings of plethysmography chamber pressure in each protocol stage are presented in Figure 1.

### Changes in Tidal Volume and Inspiratory Time

**FIGURE 2:**
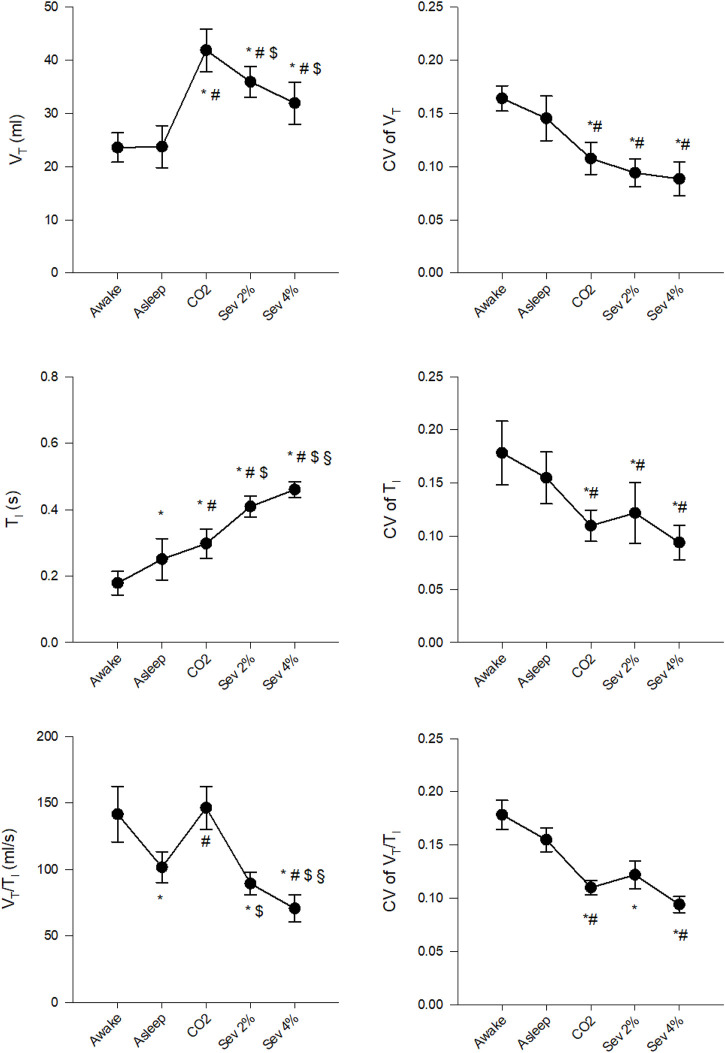
Changes in the absolute values and coefficients of variation (CV) of tidal volume (V_T_), inspiratory time (T_I_) and their ratio (V_T_/T_I_) in wakeful (Awake) and sleeping (Asleep) rabbits and during the inhalation of 5% CO_2_ (CO_2_), 2% sevoflurane (Sev 2%) or 4% sevoflurane (Sev 4%), presented as mean ± 95% confidence interval of the mean. *p < 0.05 vs. Awake, ^#^ *p* < 0.05 vs. Asleep, ^$^ *p* < 0.05 vs. CO_2_, ^§^ *p* < 0.05 vs. Sev 2%.

Changes in the mean values of V_T_, T_I_ and V_T_/T_I_ and their CVs are presented in Figure 2. After transitioning from the Awake to the Asleep stage, V_T_ did not change significantly, while T_I_ increased, resulting in a decreased V_T_/T_I_ (*p* < 0.005 for both). Compared to the Awake stage, hypercapnia increased V_T_ and T_I_ (*p* < 0.005 for both) without significant effects on V_T_/T_I_. Sevoflurane inhalation elevated V_T_ and T_I_ compared to the Awake and Asleep stages (*p* < 0.001 for all). A significantly lower V_T_/T_I_ was observed under both concentrations of sevoflurane compared to the Awake stage; however, only the Sev 4% stage differed significantly from the Asleep stage (*p* < 0.001). Similar tendencies were observed for the changes in the coefficients of variation of V_T_, T_I_ and V_T_/T_I_, with significantly lower values measured during hypercapnia and sevoflurane inhalation (*p* < 0.05 for all).

**FIGURE 3:**
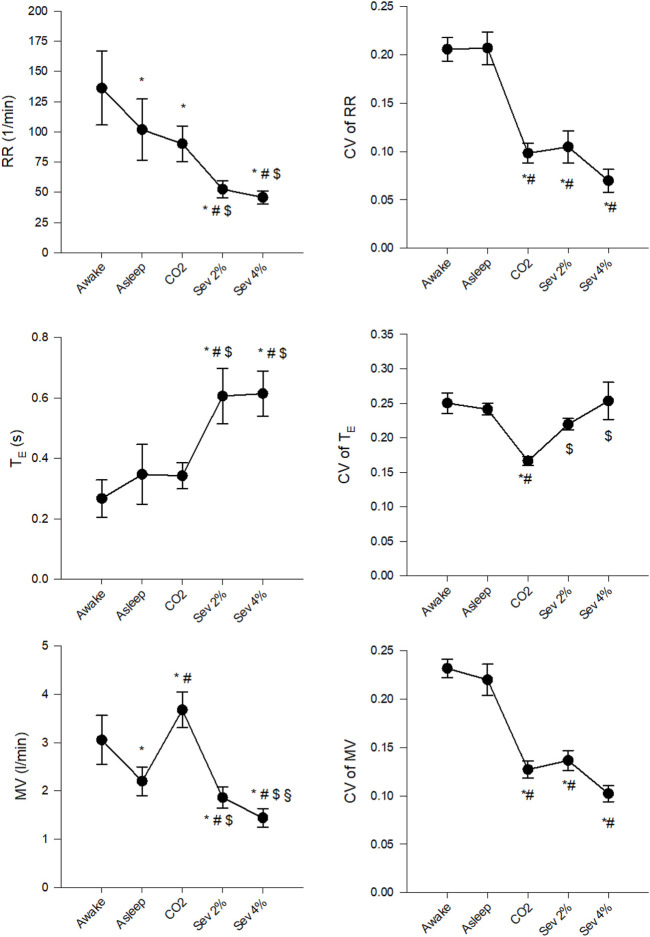
Changes in the absolute values and coefficients of variation (CV) of respiratory rate (RR), expiratory time (T_E_) and minute volume (MV) in wakeful (Awake) and sleeping (Asleep) rabbits and during the inhalation of 5% CO_2_ (CO_2_) or 2% sevoflurane (Sev 2%) or 4% sevoflurane (Sev 4%). *p < 0.05 vs. Awake, ^#^ *p* < 0.05 vs. Asleep, ^$^ *p* < 0.05 vs. CO_2_, ^§^ *p* < 0.05 vs. Sev 2%.

Figure 3 demonstrates changes in the mean values of RR, T_E_ and MV and their CVs. Significant decreases were observed in RR and MV (*p* < 0.005 for both), but not in T_E_ following the transition from the Awake to the Asleep stage. Hypercapnia decreased RR compared to the Awake stage and increased MV compared to both the Awake and Asleep stages (*p* < 0.002 for all) without significantly affecting T_E_. Sevoflurane inhalation decreased RR and MV and increased T_E_ compared to all previous stages (*p* < 0.002 for all). The CVs of RR, T_E_ and MV did not differ between the Awake and Asleep stages. Both hypercapnia and sevoflurane inhalation significantly decreased CVs of RR and MV compared to the Awake and Asleep stages (*p* < 0.001 for all), whereas, for the CV of T_E_, only the CO_2_ stage differed from the other four stages (*p* < 0.005).

### Autocorrelation Analyses

The 12 data series for each respiratory variable in each stage were categorized based on the sign (positive or negative) of their first order autocorrelation coefficients. For those variables for which the distribution of autocorrelation coefficients was dependent on protocol stage (V_T_ and T_I_), the numbers of these categorized data series are presented in Table 1. For V_T_, positive autocorrelations were less frequent in Sev 4% than in the Awake and CO_2_ stages (*p* < 0.05 for both). Furthermore, the higher concentration of sevoflurane significantly reduced the frequency of positive autocorrelation coefficients compared to the Asleep stage for T_I_ (*p* < 0.05).

**TABLE 1:** Number of data series of tidal volume (V_T_) and inspiratory time (T_I_) with positive or negative autocorrelation coefficients at lag one breath. (Sev: sevoflurane).

		Awake	Asleep	CO_2_	Sev 2%	Sev 4% ^*,#^
V_T_	Positive	9	7	10	8	1
	Negative	3	5	2	4	11
T_I_		Awake	Asleep	CO_2_	Sev 2%	Sev 4% ^§^
	Positive	10	12	10	7	5
	Negative	2	0	2	5	7

* *p*<0.05 vs. Awake.

# *p*<0.05 vs. CO_2_.

§ *p*<0.05 vs. Asleep.

### Partitioning of Total Variance Into Noise, Correlated Behavior, and Oscillatory Behavior

The relative contributions of noise, correlated behavior, and oscillatory behavior to the total variance of V_T_ and T_I_T_I_ as primary outcome variables are demonstrated in Table 2. No significant difference was found in the relative contributions of noise, correlated behavior, and oscillatory behavior to the total variance of V_T_, T_E_ or V_T_/T_I_ between the protocol stages. In comparison to the Awake and Asleep conditions, both 2 and 4% sevoflurane significantly decreased the relative contribution of correlated behavior to the variance of T_I_ (*p* < 0.05 for all). The variance attributed to oscillatory behavior did not differ between the protocol stages in any of the analyzed variables.

**TABLE 2:** Fractions of total variance of tidal volume (V_T_) and inspiratory time (T_I_) attributed to random, correlated, and oscillatory components under the different protocol stages (Sev: sevoflurane, ND: not detectable). Data are presented as mean ± 95% confidence interval of the mean.

	Source of total variance	Awake	Asleep	CO_2_	Sev 2%	Sev 4%
V_T_	Random fraction	91.7 ± 5.9%	97.8 ± 1.7%	92.5 ± 4.5%	94.5 ± 3.3%	96.7 ± 1.5%
	Correlated fraction	7.8 ± 5.9%	1.6 ± 1.1%	6.8 ± 4.3%	5.5 ± 3.3%	3.27±%
	Oscillatory fraction	0.5 ± 0.3%	0.6 ± 0.3%	0.6 ± 0.3%	ND	ND
T_I_	Random fraction	87.8 ± 6.1%	86.8 ± 4.5%	91.7 ± 5.4%	96.5 ± 2.0%	96.1 ± 3.3%
	Correlated fraction	12.17 ± 6.2%	12.22 ± 4.9%	7.6 ± 5.2%	2.7 ± 1.7%^*,#^	3.2 ± 3.0%^*,#^
	Oscillatory fraction	ND	0.1 ± 0.1%	0.1 ± 0.1%	0.1 ± 0.1%	0.1 ± 0.1%

^*^ *p* < 0.05 vs. Awake.

^#^ *p* < 0.05 vs. Asleep.

## Discussion

In the present experimental study, we investigated the changes in the variability of spontaneous breathing occurring under hypercapnia and sevoflurane. Whereas our results using the absolute values confirmed the effect of CO_2_ and sevoflurane on ventilation parameters, both interventions significantly diminished the physiological variability of V_T_, T_I_ and RR. Our data analysis revealed that an anesthetic concentration of sevoflurane decreased the frequency of occurrence of positive first order autocorrelations in V_T_ and T_I_. Furthermore, both sedative and anesthetic concentrations of sevoflurane diminished the contribution of correlated behavior to the total variance of T_I_.

### Methodological Considerations

The experiments were designed in an attempt to mimic clinical conditions encountered under sedation with hypoventilation and subsequent hypercapnia. To identify the effect of CO_2_ independent of respiratory depression caused by sedation, we administered 5% CO_2_ to the inspired gas in awake rabbits. A non-invasive measurement method was used in the present study to minimize any interference with the spontaneous breathing, since invasive measurements such as repeated arterial blood sampling, end-tidal CO_2_, electroencephalography or other monitoring apparatus would have biased the recordings of spontaneous breathing patterns. Therefore, the sleeping state achieved spontaneously after the awake condition could only be verified by visual inspection. Despite the lack of electroencephalography confirmation of the sleeping activity, several behavioral aspects of sleep in rabbits were verified through the camera feed, including body posture, depression of the ears, extension of the hindlimbs, cessation of nostril movements (Pivik et al., 1986; Aguilar-Roblero and González-Mariscal, 2020). Additionally, in accordance with 3R (Replacement, Reduction, Refinement) principles, respiratory volume calibration involving invasive ventilation was performed for only one of the rabbits, and the obtained calibration factor was applied to all animals since the box properties were constant. Thus, the results on V_T_, MV and V_T_/T_I_ were estimated with the constant calibration factor for all rabbits. It is important to note, however, that within-subject changes and indices of variability were the focus of interest in the present study, and these are not affected by the calibration factor.

The order of protocol stages was not randomized, instead, it was set so that each stage had minimal influence on the following. Sevoflurane was applied last to avoid post-anesthetic effects in the following stages. Moreover, since CO_2_ 5% stage preceded sevoflurane, the effects of hypercapnia and CO_2_ rebreathing could potentially interfere with the breathing pattern under anesthesia. To avoid such interference, a continuous fresh gas flow in the box environment was ensured and the CO_2_ concentration was continuously monitored. Even during sevoflurane stages, the CO_2_ content of the box gas was <0.3%. Thus, CO_2_ rebreathing did not affect the minute ventilation during sevoflurane stages.

### Changes in Absolute Values of Respiratory Variables

Our results for the mean absolute values obtained under spontaneous breathing are in accordance with well-established observations concerning the effects of sleep (Douglas et al., 1982a), sedation and hypercapnia on V_T_, respiratory times and rate. While V_T_ was similar between awake and asleep stages, the RR was lower during sleep. A possible explanation for these observations is anxiety-related hyperpnea of rabbits during the awake stage (Schroeder and Smith, 2011; Atalan et al., 2019) and its cessation during sleep. Furthermore, a lower metabolic rate and chemosensitivity to both oxygen (Douglas et al., 1982b) and carbon dioxide during sleep (Douglas et al., 1982c) might explain a diminished RR and MV.

Regarding the effect of hypercapnia, the significant increases in MV resulted from an altered breathing pattern, in which the markedly increased V_T_ outweighed the decreases in RR. The decreased RR is somewhat contradictory to previous results demonstrating that hypercapnia increased MV via simultaneous elevations in V_T_ and RR in humans, cats, and rats (Eckenhoff and Helrich, 1958; Read, 1967; Clark and von Euler, 1972; Peever and Stephenson, 1997; Seifert and Mortola, 2002). However, previous reports in rabbits (as well as in cats, dogs, and sheep) demonstrated that respiratory frequency can also decrease with CO_2_ concentration rising in the inspired air (Hoover et al., 1970; Jennings and Macklin, 1972; Romo-Salas et al., 1978; Maskrey and Nicol, 1980; Szlyk and Jennings, 1987). In these reports, the respiratory frequency is described to shift towards an optimal value that results in maximum CO_2_ elimination, depending on the CO_2_ concentration and the initial frequency. In our study, the respiratory rate in the Awake stage may be attributed to the initial anxiety of the animal elicited by the laboratory setting despite the 1-week acclimatization period (Schroeder and Smith, 2011; Atalan et al., 2019). The respiratory rates were comparable to those reported in a previous study in rabbits (Shafford and Schadt, 2008), in which a similarly observed rapid breathing was accompanied by normal arterial CO_2_ partial pressure (PaCO_2_, 36 mmHg). Despite this behavioral aspect, the rabbit is considered to be an excellent model for respiratory research (Keir and Page, 2008), at least with several advantages over rodents, with anatomy-physiology features that are close to humans. As such, we consider that our findings are possible to extrapolate to humans, at least in terms of the variability indices and trends.

Sevoflurane inhalation led to a dose-dependent decrease in MV, which is in agreement with experimental (Wallin et al., 1975) and clinical observations (Doi and Ikeda, 1987; Brown et al., 1998). Nevertheless, there is a discrepancy between clinical and experimental studies on the contribution of V_T_ and RR to the sevoflurane-induced changes in MV. The dominating effect of the decreased RR over the somewhat increased V_T_ observed in the present study are in accordance with previous observations in mice and dogs (Wallin et al., 1975). In contrast, sevoflurane in human subjects causes a dose-dependent decrease in V_T_ that outweighs the compensatory increase in RR (Doi and Ikeda, 1987).

### Changes in Coefficients of Variation of Respiratory Variables

As the main finding of the present study, we observed significant changes in the indices of variability of spontaneous breathing following CO_2_ and sevoflurane inhalation. In agreement with previous reports in neonates (Hathorn, 1974) and adults (Tobin et al., 1995), V_T_ and RR exhibited normal distributions, with coefficients of variation ranging between 5 and 30%, depending on the experimental stage.

Even though respiratory variability has a random component (noise) arising from external or internal sources, periodic fluctuations in RR and V_T_ do occur. Briefly, an incidental change in PaCO_2_ can be overcorrected due to the temporal delays in both the afferent and efferent limbs of the chemoreceptor feedback loop, resulting in an even greater excursion of PaCO_2_. On one hand, if the magnitude at which changes in ventilation are translated into changes in PaCO_2_ (plant gain) or the strength of the chemoreceptor response (controller gain) are high enough, sustained oscillations in ventilation can develop (Khoo, 2000). On the other hand, decrements in either gain mechanism in the feedback loop can result in a less variable breathing pattern. For instance, administration of hypercapnic gas mixtures has been shown to abolish periodic breathing (Cherniack et al., 1979) by reducing plant gain *via* decreasing the relative amplitude of variations in alveolar CO_2_ tension (Farhi and Rahn, 1955). This mechanism explains the significantly lower variability in all measured variables observed during the CO_2_ stage in the current study.

Attenuation of loop gain might also be responsible for the decreased CVs of all measured variables under sevoflurane. Although the effect of sevoflurane on loop gain (the product of plant gain and controller gain) has not yet been investigated, a previous study demonstrated the ability of another general anesthetic acting on GABA_A_ receptors, propofol, to decrease loop gain in patients with brain damage (Klimathianaki et al., 2010).

### Changes in Correlated Behavior of Respiratory Variables

Whereas first order autocorrelation was the most frequent during hypercapnia, significantly lower CVs were observed for each respiratory variable. This finding is in accordance with the enhanced positive autocorrelations in V_T_ reported in rats under hypercapnia (Khatib et al., 1991). The increased occurrence of negative autocorrelations observed under sevoflurane can be explained by the dominance of the peripheral chemoreceptor feedback loop over the central chemoreflex (Khatib et al., 1991). This imbalance between the two control mechanisms can result from the attenuation of the central chemoreflex by sevoflurane, which has been suggested to occur at deeper levels of sedation (van den Elsen et al., 1998).

## Conclusion

In summary, fundamental differences were observed in the variability of spontaneous breathing between physiological sleep and sevoflurane-induced anesthesia. While physiological sleep had no effect on the breathing variability, sevoflurane diminished markedly the fluctuations in respiratory volume, inspiratory airflow and breathing frequency. Since natural variability in tidal volume and respiratory rate facilitate alveolar recruitment and ventilation-perfusion matching, reduced breathing variability may increase the risk of atelectasis development during procedures under sevoflurane in which spontaneous breathing is maintained.

## Data Availability

The raw data supporting the conclusions of this article will be made available by the authors, without undue reservation.

## Abbreviations

ANOVA, analysis of variance; CO2, carbon dioxide; CV, coefficient of variation (ratio of the standard deviation and the mean); GABAA, gamma-aminobutyric acid type a; MV, minute volume; PaCO2, CO2 partial pressure; RR, respiratory rate; Sev 2% and Sev 4%: 2% sevoflurane and 4% sevoflurane in room air, respectively; TI, inspiratory time; TE, expiratory time; VT, tidal volume; WBP, whole-body plethysmography.
